# One-size MAP does not fit all

**DOI:** 10.1186/s13054-015-0939-0

**Published:** 2015-05-07

**Authors:** Mourad H Senussi

**Affiliations:** Respiratory Institute, Cleveland Clinic, 9500 Euclid Avenue, Cleveland, OH 44195 USA

## One-size mean arterial pressure does not fit all

The Surviving Sepsis Campaign [1] advocates maintaining a mean arterial pressure (MAP) of at least 65 mm Hg in sepsis patients undergoing resuscitation. Leone and colleagues [2], in an article published in this journal, suggest considering higher MAP targets in the resuscitation of patients with a history of arterial hypertension so they do not progress to acute kidney injury. Asfar and colleagues [3], in a multicenter, open-label trial, showed no significant difference in mortality outcomes in septic shock patients undergoing resuscitation with an MAP target of either 80 to 85 mm Hg (high-target group) or 65 to 70 mm Hg (low-target group). However, the study did show that chronic hypertensive patients in the higher-target group had lower incidences of acute kidney injury and renal replacement therapy. This likely stems from the need for higher MAPs in chronic hypertensive patients in order to maintain organ blood flow because of a shift of the organ’s autoregulatory range to the right. Thus, targeting a higher MAP for chronic hypertensive patients may help avoid the development of acute kidney injury and the need for renal replacement therapy. Renal replacement therapy carries with it inherent morbidity as well as additional cost. These costs include the need for dialysate fluid and extra personnel and the use of anticoagulation and the extracorporeal circuit [2]. However, chronic hypertensive patients in the high-target group had a greater incidence of new-onset atrial fibrillation (5.2% in the low-target group versus 9% in the high-target group). Patients with new-onset atrial fibrillation during sepsis have been shown to have increased incidences of in-hospital stroke and in-hospital mortality [4] as well as subsequent recurrence of atrial fibrillation and increased long-term risks for heart failure, ischemic stroke, and death [5]. This may offset any benefit of a higher MAP. The ideal target MAP may have to be individualized for specific patient populations. More studies are needed to determine whether baseline blood pressure plays a role in the ultimate determination of the ideal MAP target for patients with sepsis.

## Abbreviation

MAP: Mean arterial pressure
